# Exploring family support in relation to depression and nutritional status among older adults

**DOI:** 10.3389/fpubh.2026.1846159

**Published:** 2026-10-02

**Authors:** Kerime Öğüt Düzen, Gizem Helvacı

**Affiliations:** 1 Department of Gerontology, Faculty of Health Sciences, Mehmet Akif Ersoy University, Burdur, Türkiye; 2 Department of Nutrition and Dietetics, Faculty of Health Sciences, Mehmet Akif Ersoy University, Burdur, Türkiye

**Keywords:** depression, family support, geriatric health, malnutrition, nutritional status, older adults

## Abstract

**Background:**

Family support plays a key role in psychological well-being and overall health among older adults. This study investigated the relationships between family support, depression, and nutritional status, and whether family support levels vary by sociodemographic characteristics.

**Methods:**

This cross-sectional descriptive study was conducted between April and October 2025 with 281 older adults aged 65 and over living in the Mediterranean region of Türkiye, using a face-to-face interview method and a questionnaire. The questionnaire included sociodemographic characteristics, the Mini Nutrition Assessment Short-Form (MNA-SF), the Family Support Scale for the Older Adults (FSCOA) and the Geriatric Depression Scale (GDS). Data were analyzed using descriptive statistics, Kruskal-Wallis and chi-square tests, and logistic regression.

**Results:**

Depression was identified in 25.6% of the participants, while 49.5% had poor nutritional status. Statistically significant differences were observed in sex, marital status, educational status, and regular exercise status across family support groups (low,moderate,high; *p* < 0.05). Individuals in the high family support group had 67.8% lower odds of depression than those in the low group (OR = 0.322; *p* = 0.012), but family support group was not significantly associated with nutritional status (*p* > 0.05). When analyzed as a continuous variable, family support was not significantly associated with either depression or poor nutritional status (*p* > 0.05). Age and marital status were significantly associated with nutritional status and depression (*p* < 0.05). Interaction analyses showed that the relationship between family support group and both depression and nutritional status did not differ significantly by age, sex, or marital status (*p* > 0.05).

**Conclusion:**

This study showed that family support group varied significantly across sociodemographic variables. While family support was not significantly associated with nutritional status, older individuals in the high family support group had lower odds of depression compared to those in the low group. Targeted support programs may be particularly relevant for sociodemographic groups with lower levels of perceived family support, such as men and those who are widowed, divorced, or never married.

## Introduction

Family is the primary support system for maintaining the health of older adults. This support includes tangible assistance such as help with daily activities or finances, as well as regular contact and emotional encouragement from family members (1, 2). However, support has two important dimensions, perceived and received. Received support refers to the actual amount of supportive behaviors or assistance that an individual receives from others, while perceived support reflects both the individual’s satisfaction with the support they receive and their belief in the availability of support when needed (3, 4). Ensuring that older adults have both tangible support and a sense of available support substantially enhances their quality of life and their ability to cope with the demands of aging (1, 5). Nevertheless, the level of family support is influenced by a range of factors, including family structure, geographic proximity, and economic resources, all of which may change over time (6, 7).

Ensuring the mental health and overall welfare of older adults relies heavily on the emotional support and sense of belonging offered by family (8). Notably, evidence shows that emotional support yields greater benefits for reducing anxiety and depression than instrumental support. (9, 10). Family support plays an important role in supporting not only the emotional well-being of older adults, but also their healthy eating habits. Instrumental support helps with practical aspects of providing food, such as shopping or meal preparation, while emotional and relational support influences a person’s motivation and commitment to healthy eating (11). Malnutrition and depression, both significantly influenced by family support, often coexist and interact within the geriatric population. Depressive mood can lead to changes in the mechanisms regulating appetite and food intake in older adults, contributing to the development of malnutrition (12–14). Conversely, malnutrition can lead to insufficient energy intake and nutrient deficiencies, which may disrupt biological and physiological processes and increase vulnerability to depression. Dietary factors such as irregular meal timing, unhealthy dietary patterns, and inadequate nutritional balance have been associated with changes in inflammatory and oxidative processes, which may adversely influence mental health in older adults. (15, 16).

Preventing and managing psychosocial and nutrition-related health problems that arise in old age is a prominent goal in public health today. Family support is considered an important factor in addressing these challenges. Accordingly, this study aimed to examine the relationships between family support, depression, and nutritional status in older adults, as well as differences in family support levels according to sociodemographic variables. Results obtained from this study are expected to contribute to practices related to older adult health.

### Hypothesis

*H*1: Sociodemographic characteristics, health status, and regular exercise habits differ significantly by levels of perceived family support.

*H*2: Older adults in the high family support group have lower odds of depression compared to those in the low family support group, after adjusting for relevant covariates.

*H*3: Older adults in the high family support group have lower odds of poor nutritional status compared to those in the low family support group, after adjusting for relevant covariates.

*H*4: Nutritional status and depression are significantly associated with each other.

*H*5: Some sociodemographic characteristics are significantly associated with nutritional status and depression.

*H*6: The relationship between being in the high (vs. low) family support group and the odds of depression or poor nutritional status varies by age, sex, and marital status.

## Methods

This cross-sectional descriptive study was conducted from April to October 2025 among individuals aged 65 and older living in the Mediterranean region of Turkey. Participants were selected on a voluntary basis and provided written informed consent before being included in the study. Individuals living with at least one family member were included, whereas those with a history of severe psychiatric or neurological disorders or who were unable to speak vocally were excluded from the study. A total of 281 participants were included in the analyses after approximately 6% of individuals with missing data were excluded.

The participants’ sociodemographic characteristics were assessed using a data collection form developed by the researchers based on the literature. Information was collected on age, sex, marital status, place of residence, educational level, physical activity, living with family members, body weight, height, and chronic diseases. Following international standards, participants were classified as having regular physical activity (“yes”) if they performed at least 150 min of moderate-intensity or 75 min of vigorous-intensity aerobic activity per week, or an equivalent combination of both, spread throughout the week. Those below this level were classified as “no” (17). Chronic illness status was determined based on self-reported physician diagnoses of diabetes, hypertension, cardiovascular, liver, kidney, respiratory diseases, cancer, and other long-term conditions. Participants who had at least one of these diseases were classified as having a chronic illness. In addition, the survey form included three different scales: the Mini Nutritional Assessment, Family Support for the Older Adult, and Geriatric Depression Scale.

### Mini nutritional assessment short form

In assessing the nutritional status of older adults, the Mini Nutritional Assessment–Short Form (MNA-SF) was used. It consists of 6 items covering appetite changes, weight loss, mobility, psychological stress or acute illness, neuropsychological problems, and body mass index. Responses to each item are scored, and the total score is used to classify individuals’ nutritional status. The maximum possible score is 14. Based on the total score, those scoring 12–14 are considered to have normal nutrition, those scoring 8–11 are considered at risk for malnutrition, and those scoring 0–7 are considered malnourished (18).

### Family support scale for the older adults

The scale was developed by Uddin and Bhuiyan to assess the perception of family support, and its Turkish validity and reliability study was conducted by Solmaz and Aslan. The family support scale for the older adults is unidimensional and contains 20 items. The items assess 20 different areas in older adults, including respect, affection, daily activities, information, religious activities, emotional support, treatment, nutrition, personal needs, important decisions, social activities, help with problem solving, personal problems, health, important people, financial situations, sleep, friendship, happiness, and life satisfaction. The scale items are scored using a four-point Likert scale (0: Not at all, 1: A little, 2: Somewhat, 3: Very much), and the total score ranges from 0 to 60. High scores indicate that older adults perceive family support to be higher (19, 20). In this study, the level of family support was classified into tertiles based on the 33rd and 67th percentiles of the scale scores. Tertile 1 corresponds to scores of ≤52, Tertile 2 to scores of 53–55, and Tertile 3 to scores of ≥56. These cut-off points are not validated thresholds, but rather sample-derived tertiles from our study population. For clarity and ease of interpretation, these groups were referred to as “low,” “moderate,” and “high” family support, respectively, throughout the manuscript.

### Geriatric depression scale

The 15-item Geriatric Depression Scale was used to assess the existence and intensity of depression in older adults. The first version of the scale was a 30-item long form developed by Yesavage et al. in 1982 (21). The validity and reliability study of this 15-item short form, created for ease of use, was conducted by Burke et al. in 1991 (22). The validity and reliability of the scale, adapted into Turkish, were tested by Durmaz et al. in 2018. In the Turkish version, scores between 0 and 4 indicate no depressive symptoms, scores between 5 and 8 indicate mild symptoms, scores between 9 and 11 indicate moderate symptoms, and scores of 12 and above indicate severe depressive symptoms. The cutoff score is ≥5, and the risk of depression increases as the score increases (23).

### Data analysis

SPSS Statistics version 32.0 was used for the statistical analysis of the data. Descriptive analysis included the number (n), percentage, mean, and standard deviation values. Results of the Kolmogorov–Smirnov test indicated that the data did not follow a normal distribution. Therefore, the non-parametric Kruskal-Wallis test and chi-square test were used in the analysis of sociodemographic variables according to family support scale tertiles.

The participants’ nutritional status and geriatric depression distribution are presented graphically. In addition, logistic regression analyses were conducted using the presence of depression and poor nutritional status as dependent variables. Depression was categorized as “present” or “absent,” while nutritional status was categorized as “good” for participants with normal nutritional status and “poor” for those at risk of malnutrition or who were malnourished. Multicollinearity among the independent variables was assessed using VIF and tolerance statistics in SPSS. All variables in all models had VIF values below 2.5 and tolerance values above 0.4, indicating no significant multicollinearity in the data. Model fit was assessed using Nagelkerke’s R^2^ and the Hosmer-Lemeshow goodness-of-fit test.

Sample size adequacy for the depression model was assessed using the events-per-variable (EPV) approach. The EPV value, calculated with approximately 72 cases of depression and 10 parameters, was 7.2. Recognizing that a low EPV may introduce risks of model instability and overfitting, we performed internal validation using 1,000 bootstrap resamples. The bootstrap analysis demonstrated that the primary associations remained consistent in both direction and statistical significance, and that coefficient estimates closely aligned with those of the original model, supporting the stability of the regression results. Nonetheless, the potential for overfitting cannot be entirely ruled out, and the findings should therefore be interpreted with appropriate caution. For the poor nutrition model, poor nutrition was present in 139 of 281 participants, representing 49.5 percent of the sample. The model incorporated 10 independent variables, resulting in an EPV of 13.9. This exceeds the recommended cutoff of 10, indicating sufficient sample size and model stability. The significance level was set at *p* < 0.05 for all statistical analyses.

## Results

Table 1 presents the characteristics of older adults according to low, moderate, and high family support groups. The mean age was 71.2 ± 6.4 years, and the mean body mass index was 27.8 ± 3.6 kg/m^2^. Sex, marital status, educational status, and regular exercise status showed statistically significant differences between family support groups (*p* < 0.05). The proportion of women in the moderate group was higher than in the low group (*p* < 0.001). The proportion of married individuals was higher in the moderate group compared to the other two groups (*p* = 0.031). The proportion of primary school graduates is higher in the high group (*p* = 0.014). The proportion of those who do not exercise regularly is greater in the high group than in the other groups (*p* = 0.020). Age, body mass index, place of residence, living arrangement, and presence of chronic disease did not show a statistically significant difference between family support groups (*p* > 0.05; Table 1).

**Table 1:** Characteristics of the study sample according to levels of family support.

Variables	Total (*n* = 281)	Low (*n* = 84)	Moderate (*n* = 107)	High (*n* = 90)	*p* value
Age (in years)	71.2 ± 6.4	72.7 ± 7.7	70.1 ± 5.5	71.1 ± 5.8	0.096
BMI (kg/m^2^)	27.8 ± 3.6	28.0 ± 4.1	27.8 ± 3.7	27.8 ± 3.1	0.880
Sex					
Female	175 (62.3)	54 (64.3) ^a^	79 (73.8) ^b^	42 (46.7) ^b^	**<0.001**
Male	106 (37.7)	30 (35.7) ^a^	28 (26.2) ^a^	48 (53.3) ^a^	
Marital status					
Married	178 (63.3)	47 (56) ^a^	78 (72.9) ^b^	53 (58.9) ^a^	**0.031**
Single/widowed	103 (36.7)	37(44) ^a^	29 (27.1) ^a^	37 (41.1) ^a^	
Place of residence					
Urban	65 (23.1)	21 (25)	29 (27.1)	15 (16.7)	0.199
Rural	216 (76.9)	63 (75)	78 (72.9)	75 (83.3)	
Educational status					
Literate	11 (3.9)	6 (7.1) ^a^	4 (3.7) ^a^	1 (1.1) ^a,b^	**0.014**
Primary school	229 (81.5)	65 (77.4) ^a^	80 (74.8) ^a^	84 (93.3) ^b^	
Middle school	20 (7.1)	7 (8.3) ^a^	12 (11.2) ^a^	1 (1.1) ^a^	
High school and university	21 (7.5)	6 (7.1) ^a^	11 (10.3) ^a^	4 (4.4) ^a,b^	
Living arrangement					
Spouse	168 (59.8)	44 (52.4)	72 (67.3)	52 (57.8)	0.106
Children	97 (34.5)	34 (40.5)	27 (25.2)	36 (40)	
Spouse and Children	8 (2.8)	2 (2.4)	5 (4.7)	1 (1.1)	
Relatives	8 (2.8)	4 (4.8)	3 (2.8)	1 (1.1)	
Regular physical activity					
Yes	26 (9.3)	10 (11.9) ^a^	14 (13.1) ^a^	2 (2.2) ^a^	**0.020**
No	255 (90.7)	74 (88.1) ^a^	93 (86.9) ^a^	88 (97.8) ^b^	
Presence of chronic disease					
Yes	217 (77.2)	67 (79.8)	82 (76.6)	68 (75.6)	0.790
No	64 (22.8)	17 (20.2)	25 (23.4)	22 (24.4)	

Kruskal-Wallis Test and Chi-Square Test. Continuous variables are presented as mean ± standard deviation; categorical variables are presented as number (percentage). Values in the same row with different superscript letters (a, b) indicate statistically significant differences between groups (*p* < 0.05), determined by pairwise comparisons with Bonferroni correction following the chi-square test. BMI, body mass index. Bold values indicate statistically significant *p* values.

Figure 1 presents the distribution of geriatric depression and nutritional status among participants. Moderate depression was found in 0.7% of older adults, while mild depression was present in 24.9%. Depression was not detected in the vast majority of participants (74.4%). When nutritional status was evaluated, 50.5% of individuals were normal, 46.3% were at risk of malnutrition, and 3.2% were malnourished (Figure 1).

**Figure 1:**
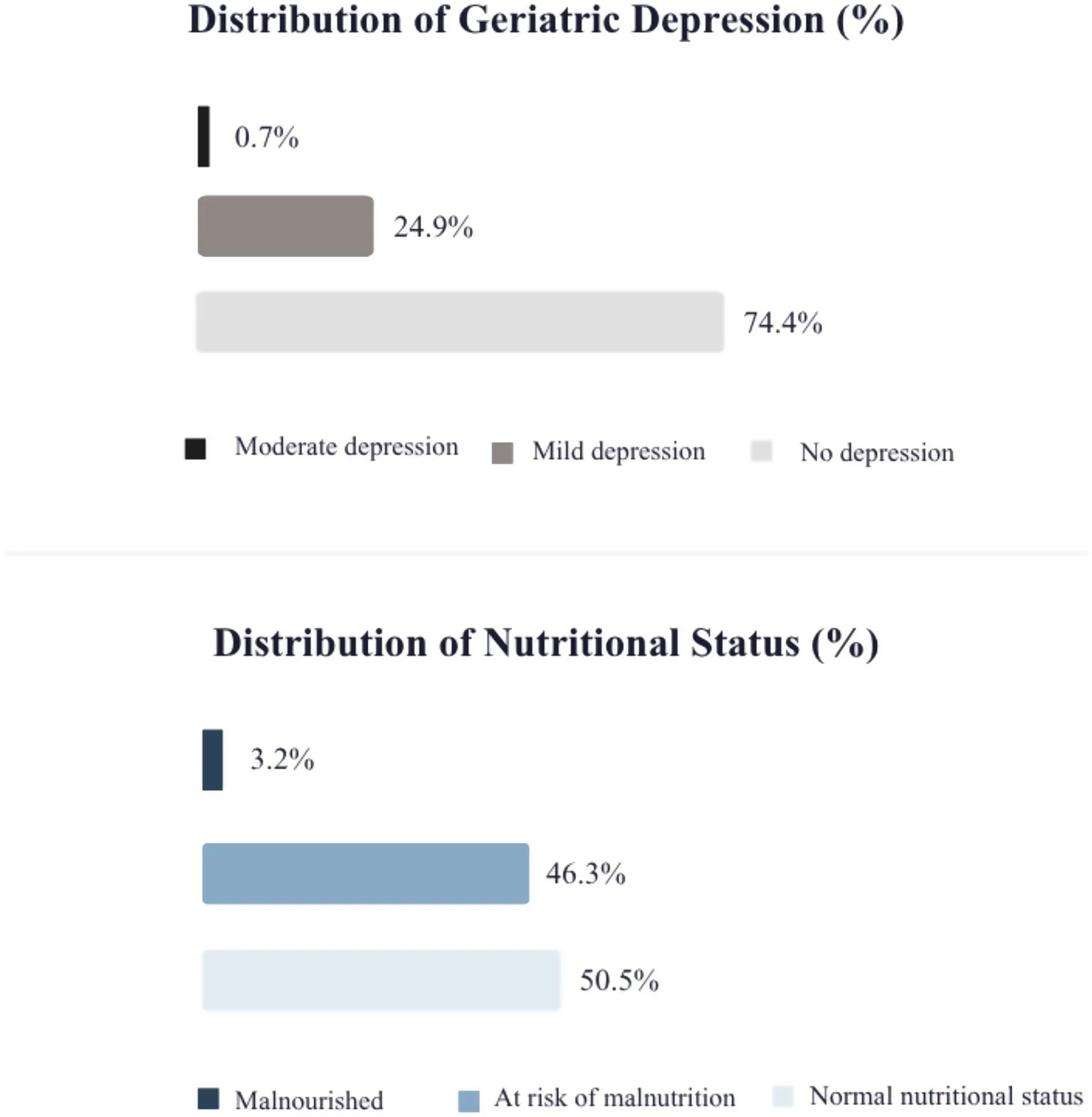
Distribution of geriatric depression and nutritional status (*n* = 281).

Table 2 presents the results of a binary logistic regression analysis examining factors associated with the presence of depression. Nagelkerke’s R^2^ value was found to be 0.165, and the analysis determined that the model was significant (*p* < 0.001). The Hosmer–Lemeshow test did not indicate a significant lack of fit (χ^2^ = 13.494, df = 8, *p* = 0.096). Each one-unit increase in age was associated with 1.112-fold higher odds of depression (95% CI: 1.042–1.187, *p* = 0.001). Married individuals had approximately 3 times higher odds of depression compared to single individuals (OR = 2.994, 95% CI: 1.196–7.497, *p* = 0.019). Participants in the high family support group had 67.8% lower odds of depression compared with those in the low family support group (OR = 0.322, 95% CI: 0.134–0.776, *p* = 0.012). Regression analysis showed that body mass index, sex, place of residence, physical activity status, presence of chronic disease, and MNA-SF score variables were not significantly associated with depression (*p* > 0.05; Table 2). Logistic regression analysis using the family support scale score as a continuous independent variable, with all other covariates held constant as in Table 2, also showed no significant association with depression (OR = 0.971, 95% confidence interval between 0.930 and 1.015, *p* = 0.192). This additional analysis is not presented in the table.

**Table 2:** Binary logistic regression results with depression presence as the dependent variable.

Covariates	B	SE	Wald	*p* value	OR	95%CI	
						LL	UL
Age	0.106	0.033	10.171	**0.001**	1.112	1.042	1.187
BMI	0.024	0.041	0.338	0.561	1.024	0.945	1.110
Sex	0.189	0.322	0.343	0.558	1.208	0.642	2.271
Marital status	1.097	0.468	5.487	**0.019**	2.994	1.196	7.497
Place of residence	0.447	0.341	1.719	0.190	1.563	0.802	3.050
Regular Physical Activity	−0.390	0.448	0.756	0.384	0.677	0.282	1.630
Presence of Chronic disease	−0.014	0.362	0.001	0.970	0.986	0.485	2.006
MNA-SF total score	0.073	0.092	0.628	0.428	1.076	0.898	1.288
Family support level							
Low (reference)							
Moderate vs Low	0.466	0.332	1.974	0.160	1.594	0.832	3.055
High vs. Low	−1.132	0.448	6.376	**0.012**	0.322	0.134	0.776

Nagelkerke’s R^2^ = 0.165; *p* < 0.001. Hosmer–Lemeshow test χ^2^ = 13.494; df = 8; *p* = 0.096. B, regression coefficient; SE, standard error; OR, odds ratio; CI, confidence interval; LL, lower limit; UL, upper limit; BMI, body mass index; MNA-SF, Mini Nutritional Assessment-Short Form. Bold values indicate statistically significant *p* values.

Table 3 presents the results of a binary logistic regression analysis conducted to examine factors associated with nutritional status. Nagelkerke’s R^2^ value was found to be 0.126, and the analysis determined that the model was significant (*p* < 0.05). The Hosmer–Lemeshow test did not indicate a significant lack of fit (χ^2^ = 8.056, df = 8, *p* = 0.428). A one-unit increase in age was associated with 1.071-fold higher odds of poor nutritional status (95% CI: 1.008–1.137, *p* = 0.026). A one-unit increase in body mass index was associated with 9.7% lower odds of poor nutritional status (OR = 0.903, 95% CI: 0.840–0.970, *p* = 0.005). Married participants had 2.2-fold higher odds of poor nutritional status compared with single participants (OR = 2.208, 95% CI: 1.014–4.808, *p* = 0.046). Regression analysis showed that the variables of sex, place of residence, physical activity status, presence of chronic disease, geriatric depression scale score, and family support level were not significantly associated with nutritional status (*p* > 0.05) (Table 3). Logistic regression analysis using the family support scale score as a continuous independent variable, with all other covariates held constant as in Table 3, also showed no significant association with poor nutritional status (OR = 0.980, 95% confidence interval between 0.941 and 1.021, *p* = 0.338). This additional analysis is not presented in the table.

**Table 3:** Binary logistic regression results with nutritional status (good vs. poor) as the dependent variable.

Covariates	B	SE	Wald	*p* value	OR	95%CI	
						LL	UL
Age	0,069	0,031	4,981	**0,026**	1,071	1,008	1,137
BMI	−0,102	0,037	7,795	**0,005**	0,903	0,840	0,970
Sex	0,365	0,271	1,815	0,178	1,440	0,847	2,448
Marital status	0,792	0,397	3,979	**0,046**	2,208	1,014	4,808
Place of residence	−0,519	0,313	2,740	0,098	0,595	0,322	1,100
Regular Physical Activity	0,307	0,444	0,480	0,488	1,360	0,570	3,245
Presence of Chronic Disease	0,362	0,315	1,324	0,250	1,436	0,775	2,661
Geriatric depression scale total score	−0,006	0,087	0,005	0,942	0,994	0,838	1,179
Family support level							
Low (reference)							
Moderate vs. Low	−0,173	0,312	0,309	0,578	0,841	0,456	1,549
High vs Low	−0,514	0,330	2,430	0,119	0,598	0,313	1,141

Nagelkerke’s R^2^ = 0.126; *p* < 0.05. Hosmer–Lemeshow test χ^2^ = 8.056; df = 8; *p* = 0.428. B, regression coefficient; SE, standard error; OR, odds ratio; CI, confidence interval; LL, lower limit; UL, upper limit; BMI, body mass index. Bold values indicate statistically significant *p* values.

Table 4 displays the results of logistic regression analyses assessing the interaction of family support (high vs. low) with various variables in relation to depression and nutritional status. The relationship between depression status and family support (high vs. low) did not differ significantly according to age, sex, marital status, and MNA score (*p* > 0.05). Similarly, no significant differences were observed in the relationship between nutritional status and family support (high vs. low) when considering age, sex, marital status, and depression status (*p* > 0.05). Furthermore, when family support was analyzed as a continuous variable, the results remained unchanged, with no significant interaction effects observed (not shown in the table).

**Table 4:** Interaction analysis.

Outcome variable	Interaction terms	B	SE	OR	95%CI	*p* value
Depression	age * family support (high vs. low)	−0.122	0.069	0.885	0.773–1.013	0.076
	sex * family support (high vs. low)	1.055	0.842	2.871	0.551–14.954	0.210
	marital status * family support (high vs. low)	0.055	0.840	1.057	0.204–5.485	0.948
	MNAscore* family support (high vs. low)	−0.342	0.355	0.711	0.355–1.424	0.336
Nutritional status	age * family support (high vs. low)	−0.047	0.045	0.954	0.872–1.043	0.297
	sex * family support (high vs. low)	−0.245	0.563	0.783	0.260–2.357	0.663
	marital status * family support (high vs. low)	−0.407	0.560	0.665	0.222–1.995	0.467
	Depression * family support (high vs. low)	0.235	0.212	1.265	0.835–1.917	0.268

All logistic regression models are adjusted for age, sex, marital status, place of residence, regular exercise, chronic disease, body mass index, MNA score, and depression score, except for variables included as part of the interaction term. B, regression coefficient; SE, standard error; OR, odds ratio; CI, confidence interval.

## Discussion

This study tested several hypotheses regarding the associations between family support group, depression, and nutritional status in older adults. Among these, H1, H2, and H5 were supported. Family support group differed according to certain sociodemographic characteristics and physical activity(H1), older adults in the high family support group had lower odds of depression compared to those in the low family support group (H2), and both nutritional status and depression were associated with some sociodemographic characteristics (H5). In contrast, H3, H4, and H6 were not supported. Being in the high family support group was not significantly associated with poor nutritional status (H3), there was no significant association between nutritional status and depression (H4), and interaction analyses did not reveal significant moderating effects of age, sex, or marital status (H6).

In this study, 25.6% of participants had depression, while 49.5% had malnutrition risk or malnutrition. In studies conducted in Turkey, higher rates of depression are generally reported (46.2, 51.8, and 56.5%), while values close to our results can also be found in the literature (24 and 24.4%) (24–27). In addition, the depression found in our study was largely mild. The relatively low rate and level of depression may be due to the fact that all participants lived with at least one family member and thus benefited from constant social and emotional support. In this study, the rates of malnutrition or risk of malnutrition according to the MNA assessment (49.5%) were higher than the rates reported in other studies conducted in the same country (26.9–42%) (28, 29). The rate in our study is close to the rates among outpatients (39.3–45.8%) but lower than the rates among inpatients (60–82.6%) (30–33). The high rate of chronic disease in our sample (77%) may have contributed to the total rate of malnutrition and risk of malnutrition reflecting the outpatient group rather than the general population.

Analysis of socio-demographic variables revealed that levels of family support showed significant differences based on sex, marital status, educational status, and physical activity. The results of the present study, which found that women’s perception of family support was higher than that of men, are consistent with literature indicating sex-related differences in the type and perception of social support (34, 35). While our study did not measure the underlying mechanisms, previous research has suggested that socialization experiences and sex-related social roles may contribute to these differences (34). In our study, the group with a medium level of perceived family support had a higher proportion of married individuals. This result may indicate that married individuals tend to perceive family support at moderate levels, leading to more balanced perceptions. Our findings align with previous studies that have reported a relationship between social support and marital status in older adults. Spouses are often reported as the most prominent source of social and family support among older adults (36, 37). However, it is possible that different sources of support, such as children, grandchildren, and relatives, also play a role in shaping perceptions of family support, and that individuals’ relationships with other family members may vary according to marital status. In line with this, a study has reported that married individuals perceive more social support from their children (38).

Looking at the demographic variables, the proportion of individuals with a primary school education was found to be high in the high family support group. The literature has associated low educational attainment in older adults with emotional and social isolation (39, 40). Our result, which appears to contradict the literature, may be interpreted as primary school graduates perceiving more family support because they have fewer sources of social support outside the family and a relatively lower level of independence. However, this is only a possible explanation and was not directly examined in our study. In our study, it was observed that the proportion of individuals who did not exercise regularly was higher in the group with the highest level of family support. A systematic review indicated that social support for physical activity is effective when it comes from family, while no relationship was found with support from friends (41). Other studies have also reported that older adults supported by their families have increased levels of physical activity (42, 43). The literature particularly emphasizes the effectiveness of family support for physical activity. However, in our study, general family support was evaluated rather than support specifically for physical activity, which may explain the differing results. It is also possible that the inverse relationship observed in our study relates to the fact that older adults with more health problems receive higher family support while being less likely to participate in physical activity. Further research is needed to clarify these relationships.

In logistic regression analysis, age, marital status, and family support were found to be associated with geriatric depression. Each one-year increase in age was associated with approximately 11% higher odds of depression. Although the association per one-year increase in age was relatively modest, the difference in odds may become more pronounced across larger age intervals. This finding suggests that age may be an important factor to consider when identifying older adults who may benefit from closer assessment of depressive symptoms and appropriate psychosocial support. Numerous meta-analyses have examined the relationship between age and depression risk. One of these studies reported that being over 75 years old, and another said that being over 80 years old, constitutes a strong risk factor for depression (44, 45). Another meta-analysis showed that getting older is a big risk factor for depression in people over 80, but this wasn’t true for people over 85 (46). A large-scale prospective cohort study found that older adults with a biological age more advanced than their chronological age had a significantly higher risk of developing depression (47). Our results showed that married individuals had approximately threefold higher odds of experiencing depression compared to single individuals. Prior research has indicated that divorced, widowed, and never-married older adults constitute a high-risk demographic for depressive symptoms (48, 49). According to a meta-analysis, compared to married older adults, the risk of depression was 1.7 times higher in widowed individuals, 2.1 times higher in divorced individuals, and 1.3 times higher in never-married individuals (50). Previous studies have discussed factors such as economic security, emotional support, and social belonging in relation to marital status and depression (51, 52). Therefore, the possible reason for these opposite results may be the complexity of family and marital relationships, including factors such as interpersonal conflict, caregiving responsibilities, or perceived loss of autonomy.

In regression analysis, being in the high family support group was associated with 67.8% lower odds of depression compared with being in the low family support group. This finding may have clinical and public health relevance, as depression was observed in 10% of the high family support group compared with 28.6% of the low family support group. Importantly, the association between family support and depression differed between the categorical and continuous analyses. This may partly reflect the use of sample-specific tertile cut-offs, which may have emphasized differences between the groups. From a clinical perspective, the categorical findings may suggest that low family support could help identify older adults who may benefit from closer psychosocial assessment. Family-based interventions that encourage family members to support the psychological well-being of older adults may be considered as supportive approaches. However, family support group was not found to be significantly associated with nutritional status. The current literature highlights that family support may have a positive effect on both conditions (53–55). Family support has been reported to play a facilitating role in both preventing geriatric depression and coping with existing depression, while insufficient support may exacerbate negative feelings (56). Nutrition studies have indicated that in the absence of family support, individuals may be at increased risk of food insecurity, their diet quality may be negatively affected, and their health status may deteriorate (57, 58). In addition to the presence of family support, the level of knowledge and attitudes of families about nutrition may also influence the nutritional status of older adults. This is because the support provided by families can include not only food provision but also motivating older adults about nutrition and encouraging them to maintain dietary patterns appropriate to their needs (53). The lack of a significant association in our study may be related to several factors. These include economic conditions, health status, and families’ knowledge and attitudes regarding nutrition, as well as the possibility of unmeasured confounding variables. Although validated measurement tools and an adequate sample size were used in this study, it remains possible that other factors not captured in our analysis influenced the results. Future studies with broader assessments and consideration of additional variables are needed to further clarify the relationship between family support and nutritional status.

This study revealed that age and marital status were significantly associated with nutritional status. It was found that each additional year of age was associated with a 7.1% higher risk of poor nutritional status. Although the magnitude of the association between age and poor nutritional status was lower than that observed for depressive symptoms, the difference in odds may become more pronounced across larger age intervals. These findings highlight the need for closer monitoring of nutritional status and depressive symptoms, particularly among older adults of advanced age. This result aligns with previous studies, which have suggested that changes in digestion, absorption, and appetite associated with aging may contribute to increased risk of malnutrition (59–61). Married individuals had 2.2-fold higher odds of poor nutritional status than single individuals. Conversely, the literature generally reports that married older adults have a lower risk of malnutrition (62, 63). The findings of this study may reflect differences in meal frequency, dietary diversity, appetite, food intake, and access to nutritionally adequate meals between married and unmarried older adults. Considering that all single individuals in our study lived with other family members, they may have received better nutritional care and support compared to married individuals. However, as the relationship between marital status and nutritional status is complex and may be influenced by many factors (such as meal preparation responsibility, food access, family stressors, and shared mealtimes), further research is warranted to clarify these associations.

Previous research has shown a bidirectional relationship between malnutrition and depression in older adults (64, 65). Poor diet quality and unbalanced eating patterns have been linked to an increased risk of depressive symptoms (66, 67). Dietary interventions, particularly those involving healthy dietary patterns tailored to individual health needs, have also been shown to be beneficial in reducing depressive symptoms in this population (68, 69). Conversely, depressive symptoms in older adults are a major contributor to reduced appetite and diminished motivation to eat, potentially creating a vicious cycle in which malnutrition and depression mutually exacerbate one another (70). Although changes in appetite-related hormones and gut microbiota composition may play a role, the precise cellular and metabolic mechanisms involved remain uncertain (66, 67). This study did not identify a possible association between depression and malnutrition. Since both conditions are related to numerous biological, clinical, psychosocial, and environmental factors, the statistical significance, strength, and direction of their relationship may vary across different samples. As such, specific characteristics of the sample, including a lower prevalence of depressive symptoms, higher rates of poor nutritional status, and the absence of individuals living alone, may help contextualize the lack of association observed between depression and malnutrition.

### Study limitations

The study has some limitations. First, the data used in the study were collected through questionnaires based on the participants’ subjective opinions, which increases the risk of information bias. Second, due to the cross-sectional nature of the study, no causal relationship could be established between the variables. Third, although age, BMI, sex, marital status, place of residence, regular physical activity, and presence of chronic disease were included as covariates in the regression analysis, other potential confounding variables such as income, cognitive status, medication use, functional status, social isolation, frailty, and tobacco or alcohol consumption could not be included due to lack of available data. This may have influenced the observed associations. Fourth, the study did not include an *a priori* sample size calculation. Although the final sample size was relatively adequate, particularly for the nutritional status model, the number of events per variable for the depression model was somewhat lower. This lower number of events per variable may have increased the risk of model instability and limited the detection of small or modest associations. Fifth, only older adults living with at least one family member were included in the study, which introduces a risk of selection bias. Therefore, this significantly limits the generalizability of the findings to older adults living alone, who may represent the population at highest risk for low family support, depression, and malnutrition. Finally, as the scope of the study was limited to the Mediterranean Region and older adults living in the community, the results cannot be generalized to older adults living in different geographical regions and receiving institutional care. It is recommended that the study be repeated in different regions, with broader participation and a longitudinal design, and with the addition of qualitative methods.

## Conclusion

This study found that the extent to which individuals perceive family support varies across sociodemographic groups. The high family support group was significantly associated with lower odds of depression compared with the low family support group, whereas no significant relationship was observed when family support was analyzed as a continuous variable. No significant association was found between family support and nutritional status in either group-based or continuous analyses. The relationship between family support group and depression did not differ significantly by age, sex, or marital status. Public health policies and clinical interventions targeting older adults should consider not only individual treatment approaches but also strategies that involve family and social support. Targeted support programs may be particularly beneficial for sociodemographic groups with lower levels of perceived family support, such as men and those who are widowed, divorced, or never married.

## Data Availability

The raw data supporting the conclusions of this article will be made available by the authors, without undue reservation.
